# Management of peri-implantitis: a systematic review, 2010–2015

**DOI:** 10.1186/s40064-016-1735-2

**Published:** 2016-02-01

**Authors:** Nisha Mahato, Xiaohong Wu, Lu Wang

**Affiliations:** Department of Prosthodontics, Stomatological Hospital of Chongqing Medical University, No. 426 Songshi Bei Road, Yubei District Chongqing, 401147 People’s Republic of China; Chongqing Key Laboratory for Oral Diseases and Biomedical Sciences, No. 426 Songshi Bei Road, Yubei District Chongqing, 401147 People’s Republic of China; Chongqing Municipal Key Laboratory of Oral Biomedical Engineering of Higher Education, No. 426 Songshi Bei Road, Yubei District Chongqing, 401147 People’s Republic of China

**Keywords:** Peri-implantitis treatment, Surgical and non-surgical therapy, Dental implant bone loss

## Abstract

Peri-implantitis or Periimplantitis is characterized as an inflammatory reaction that affects the hard and soft tissue, which results in loss of supporting bone and pocket formation surrounding the functioning osseointegrated implant. This review aimed to evaluate the effectiveness of surgical and non-surgical treatment of peri-implantitis. The data sources used was PubMed. Searches of this database were restricted to English language publications from January 2010 to June 2015. All Randomized Controlled Trials describing the treatments of peri-implantitis of human studies with a follow up of at least 6 months were included. Eligibility and quality were assessed and two reviewers extracted the data. Data extraction comprised of type, intensity provider, and location of the intervention. A total of 20 publications were included (10 involving surgical and 10 involving non-surgical mechanical procedure). The non-surgical approach involves the mechanical surface debridement using carbon or titanium currettes, laser light, and antibiotics whereas, surgical approach involves implantoplasty, elevation of mucoperiosteal flap and removal of peri-inflammatory granulation tissue followed by surface decontamination and bone grafting. This study reveals that non-surgical therapy tends to remove only the local irritant from the peri-implantitis surface with or without some additional adjunctive therapies agents or device. Hence, non-surgical therapy is not helpful in osseous defect. Surgical therapy in combination with osseous resective or regenerative approach removes the residual sub-gingival deposits additionally reducing the peri-implantitis pocket. Although there is no specific recommendation for the treatment of peri-implantitis, surgical therapy in combination with osseous resective or regenerative approach showed the positive outcome.

## Background

Implant based dental rehabilitation technique has come to offer steadfast result hence it has become a cardinal entrenched therapy in order to restore missing natural teeth in regular clinical practice. van Velzen et al. (2014) has reported 91.6 % of success rate for dental implant and shows 7 % of peri-implantitis after 10 years follow up. Dental implant has majority of success rate in long term however failure does occur. Peri-implant disease which is commenced by bacteria have two subtypes (1) Peri-implant mucositis and (2) Peri-implantitis. Peri-implant mucositis is the reversible inflammatory process of the soft tissue surrounding the peri-implant, which is followed by reddening, swelling and bleeding on probing (Mombelli et al. 2012).

Peri-implantitis or Periimplantitis is characterized as an inflammatory reaction that affects the hard and soft tissue, which results in loss of supporting bone and pocket formation surrounding the functioning osseointegrated implant (McCrea 2014). Peri-implantitis has been put under three categories depending on the pocket depth and bone loss (Table 1) (Froum and Rosen 2012).

**Table 1:** Classification of peri-implantitis (Froum and Rosen 2012)

Early	PD ≥4 mm (bleeding and/or suppuration on probing)^a^ Bone loss <25 % of the implant length^b^
Moderate	PD ≥6 mm (bleeding and/or suppuration on probing)^a^ Bone loss <25–50 % of the implant length^b^
Severe	PD ≥8 mm (bleeding and/or suppuration on probing)^a^ Bone loss >50 % of the implant length^b^

^a^Noted one two or more aspects of the implants

^b^Measured on radiographs from time of definitive prosthesis loading to current radiograph. If not available, the earliest available radiograph following loading should be used

Implant failure could be due to imbalanced occlusal force, smoking habit, poor bone quality, implant thread design, improper surgical placement, surgical trauma, incorrect prosthetic design, poor oral hygiene, bacterial infection, diabetes, the particles released from implant, etc. Bacterial infection is considered as the most important factor for implant failure. Microbiota associated with peri-implantitis are *Prevotella intermedia*, *Porphyromonas gingivalis*, *Aggregatibacter actinomycetemcomitans*, *Bacterioides forsythus*, *Treponema denticola*, *Prevotella nigrescens*, *Peptostreptococcus micros*, *Fusobacterium nucleatum*, etc. (Ata-Ali et al. 2011).

Peri-implantitis is latent in early stage and usually diagnosed during routine dental check up. Hence early diagnosis of peri-implantitis is very important to terminate the further progression of the diseases and for establishment of good osseointegration. Various treatment modalities have been put forward for the treatment of peri-implantitis, which are summarized in two treatment methods, namely resective and regenerative therapies. Resective implant treatment attempts to eliminate the etiologic factors and maintain optimal peri-implant conditions, mainly by cleaning the surfaces of the implants; whereas regenerative periodontal therapy (using bone grafts, membranes and growth factors) aims to regenerate a new attachment apparatus and reconstruct the periodontal unit to previously existing normal physiologic limits (Kim et al. 2011; Smeets et al. 2014). An optimal objective of peri-implantitis management should be the eradication of the diseases (no bleeding on probing, no further bone loss) and formulation of hard and soft peri-implant tissue. This review aims to evaluate the ideal surgical treatment of peri-implantitis in humans in a broader way than previous studies.

The aim of the present study is to assess the effectiveness of treatment of peri-implantitis.

## Review

### Rationale and focused question

To our knowledge from indexed literature, there is no absolute explanation regarding the effectiveness of surgical and non-surgical management of peri-implantitis.

The addressed focused question is: “What is the recommended treatment for management of peri-implantitis?”

### Search methods to identify relevant studies (Table 2)

An electronic search of database PubMed was conducted. Searches were limited to studies involving humans, in English language and published from January 2010 to June 2015. A random combination of following terms was used for the search: “peri implantitis treatment”, “bone grafting peri implantitis”, “therapy peri implantitis”, “dental implant inflammation”, and “dental implant bone Loss”. All retrieved articles were reviewed to identify additional relevant RCTs. The titles and abstracts of potential references were manually examined to exclude irrelevant publications, and two reviewers for additional pertinent studies reviewed all of the remaining literatures on the topic of interests independently.

**Table 2:** Systematic search strategy

Focus question	What is the recommended treatment for management of peri-implantitis?
*Search strategy*	
Population	Patients diagnosed with peri-implantitis
Intervention or Exposure	Treatment
Comparison	Non-surgical treatment with surgical treatment
Outcome	Resolution of disease: implant survival and absence of PD ≥4 mm with suppuration/BoP and no further bone loss
Search keywords	Peri-implantitis treatment, bone grafting peri-implantitis, therapy peri-implantitis, dental implant bone loss, dental implant inflammation
*Database search*	
Electronic	PubMed
*Selection criteria*	
Inclusion criteria	Include patients with at least one dental osseointegrated implant affected by peri-implantitis Describe a clinical intervention aiming at the treatment of the condition Describe a pathological condition of peri-implantitis with bone loss Experimental human studies Full-text articles (Randomized and Controlled Clinical Trials) Follow up of at least 6 months
Exclusion criteria	No access to an English version of title and abstract

### Eligibility criteria

The following eligibility criteria were imposed: (1) Original articles; (2) Experimental human studies; (3) Reference list of pertinent original and review studies; (4) Intervention: Effectiveness of peri-implantitis after surgical and non-surgical treatment; (5) Articles published only in English-language; and (6) Full-text articles (Randomized and Controlled Clinical Trials). Letters to the editor, historic reviews, abstract with no full text articles and unpublished articles were excluded.

### Data extraction and quality assessment

All datas from the eligible studies were extracted by two independent reviewers with a predefined table (Table 3). Data tables were designed to extract all relevant data from texts, tables and figures, including author, year, implant number, treatment method, duration of follow up and the outcomes.

**Table 3:** Surgical and non-surgical experimental studies of dental implants following treatment of peri-implantitis

References	Diagnosis of peri-implantitis	No. of implant	Treatment strategy		Follow up	Study parameters	Results
			Group 1	Goup 2			
Schar et al. (2013)	PPD 4–6 mm BOP Bone loss—0.5–2 mm No mobility	67	Photodynamic therapy	Minocycline Microspheres locally	6 months	BOP CAL PPD Mucosal recession Modified plaque index	Both treatment equally effective but no complete resolution of inflammation
Schwarz et al. (2006b)	PD 4 mm BOP Suppuration No mobility Keratinized peri-implant mucosa	12	Er:YAG laser		6 months	Plaque index BOP PD Gingival recession CAL Histo-pathology	Improved clinical parameters Mixed chronic inflammatory cell infiltrate
Renvert et al. (2006, 2008)	PD ≥4 mm Bleeding/pus on probing Bone loss ≤1.8 mm Anaerobic bacteria	95	Minocycline microspheres locally 1 mg	1 % chlorhexidine gel	12 months	PD BOP Local plaque index Bonel level Bacterial count	Both treatment resulted marked reduction in indicator bacteria Minocycline treatment improved PD
Persson et al. (2010)	PPD ≥4 mm Bone loss >2.5 mm Bleeding/pus on probing	–	Curettes	Ultrasonic device	6 months	PD BOP Bacterial count	Both methods failed to eliminate bacterial counts
Hallstrom et al. (2012)	PPD ≥4 mm Bleeding/pus on probing	–	Non-surgical debridment Systemic antibiotics	Non-surgical debridment	6 months	PD BOP Bacterial count	BOP and PPD were improved with antibiotic treatment No changes in bacterial count in both groups
Sahm et al. (2011)	PPD ≥4 mm Bone loss ≤30 % Bleeding Suppuration No mobility No occlusal overload 2 mm keratinized attached mucosa Good PI	43	OHI (Oral Hygiene Instructions) Amino acid glycine powder (AAD)	Mechanical debridement with carbon curettes Antiseptic therapy chlorhexidine digluconate (MDA)	6 months	BOP PD CAL	Both groups revealed comparable PD reduction and CAL gains Higher changes in BOP in AAD group
Renvert et al. (2011), and Persson et al. (2011)	PPD ≥5 mm Bone loss ≥2 mm BOP	100	Er:YAG laser	Air-abrasive device	6 months	PPD BOP Bacterial counts	Both method showed limited clinical improvement but failed to reduce bacterial count.
Karring et al. (2005)	PPD ≥5 mm Bone loss ≥1.5 mm BOP	–	Vector^**®**^ system	Submucosal debridment with carbon fiber curette	6 months	Plaque BOP PPD Bone level	There was no significant difference between the two methods although BOP was reduced in Vector^**®**^ system
Machtei et al. (2012)	PD 6–10 mm BOP Bone loss	77	Implant debridement Matrix chips (MatrixC)	Implant debridement Chlorhexidine chips (PerioC)	6 months	BOP PD CAL	PerioC showed greater clinical improvement than MatrixC
Aghazadeh et al. (2012)	PD ≥5 mm BOP Bone loss ≥3 mm Suppuration Mucosal recession	75	Resection surgery Autogenous bone Collagen membrane Antibiotic	Resection surgery Bovine derived xenograft (BDX) Collagen membrane Antibiotic	12 months	PD BOP Suppuration Bone loss	BDX with collagen membrane showed more radiographic bone defect fill Bothe treatment offered improvement in BOP, PD and suppuration
Schwarz et al. (2008, 2009)	PD >6 mm Bone loss >3 mm Keratinized mucosa	22	Access flap surgery Hydroxy-apatite nanocrystals	Access flap surgery Natural bone mineral Collagen membrane	24 months and 4 years	Plaque BOP PPD Bone level Attachment loss	Natural bone plus membrane offered better result
Schwarz et al. (2006a)	PD >6 mm Bone loss >3 mm Keratinized mucosa	22	Access flap surgery Hydroxy-apatite nanocrystals	Access flap surgery Bovine derived xenograft Collagen membrane	6 months	Plaque BOP PPD Bone level Attachment loss	Both treatment offered PD reduction and CAL gain
Wohlfart et al. (2012)	PD ≥5 mm Bone loss ≥4 mm BOP	32	Resective surgery using titanium curettes 24 % ethylenediaminetetraacetic acid	Resective surgery using titanium curettes Porous titanium granules (PTG)	12 months	PPD Bone level BOP	Reconstruction with PTG resulted better radiographic peri-implant defect fill
Romeo et al. (2007)	PD ≥4 mm Bleeding Suppuration No implant mobility Radiographic horizontal peri-implant radiolucency	38	Amoxicillin 50 mg/kg prior to treatment for 8 days Implantoplasty	Amoxicillin50 mg/kg prior to treatment for 8 days Resective surgery	3 Years	Marginal bone loss	Radiographs revealed implantoplasty as an effective treatment
Schwarz et al. (2011, 2012)	PD >6 mm Intrabony defect >3 mm- 2 mm Keratinized mucosa	26 and 38	Resective surgery Implantoplasty Er:YAG laser in intra bony components Natural bone Mineral Collagen membrane	Resective surgery Implantoplasty Cotton pellets dipped in sterile saline (CPS) Natural bone mineral Collagen membrane	6 and 24 months	BOP Attachment loss Bone loss	24 months treatments with CPS offered better clinical parameters as well as bony defect fill
de Waal et al. (2013)	PD ≥5 mm Bone defect ≥2 mm Bleeding Suppuration	79	Resective surgery with apically repositioned flap Bone recountouring Surface debridment/decontamination 0.12 % chlorhexidine 0.05 % cetylpyrinidium chloride (CPC)	Placebo	12 months	BOP Suppuration PD Bacterial count	CHX + CCP treatment results immediate suppression of bacterial count

*PPD* periodontal pocket depth, *PD* pocket depth, *BOP* bleeding on probing, *CAL* clinical attachment loss

### Study selection

**Fig. 1:**
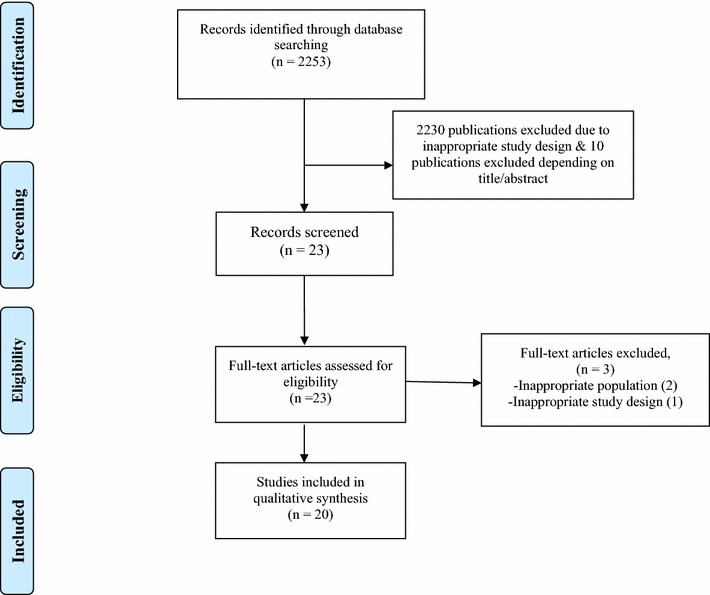
PRISMA flow chart

At each stage of the study screening, two reviewers independently reviewed the studies and made selections for inclusion (Fig. 1). All selected studies were screened by title and abstract, and the full texts of the relevant papers were then reviewed.

### Statistical analysis

A meta-analysis of trial data was not possible due to heterogeneity in trial design and outcomes reported. Data related to trial quality was therefore subject to narrative synthesis. Trial quality was assessed using the Critical Appraisal Skills Programme and PRISMA-2009 Checklist.

### Risk of bias included in studies

There could be potential language bias in this systematic review as we only considered literature written in English.

## Results

### Search results

Using the search strategy above, 2253 articles were retrieved. After reviewing title and abstracts, 2230 of those articles were excluded and 23 studies were included because the focus of this review is randomized controlled trials on therapy of peri-implantitis (Fig. 1). Among 23 studies, we excluded 2 because these 2 studies did not meet the criteria to diagnose peri-implantitis and another 1 study which failed to meet the criteria of at least 6 month follow up. In total, 20 articles were included in this review. The pattern of the current review was customized to mainly summarize the pertinent information.

## Description of eligible studies

### Treatment of peri-implantitis

Bio-film and bacteria on the surface of implant plays an important role in the appearance of peri-implantitis (Canullo et al. 2015). The management of peri-implantitis is focused on infection and bacterial controls. The treatments proposed for peri-implant disease are based on the evidence gained from the treatment of periodontitis. Both surgical and non-surgical techniques have been developed for the treatment of peri-implantitis.

#### Non-surgical techniques

The treatment of peri-implantitis in the case of incipient bone loss involves the elimination of local irritants with or without surface decontamination, systemic antibiotics, some additional adjunctive therapies agents or devices (Machtei 2014).

In the articles included in our review (Table 3), a total of 730 patients were treated with a follow up period of 6 months to 4 years with a pocket depth of >4 mm, radiological confirmed bone loss of ≥1.5 mm, exposed implant thread, absence of mobility and the presence of bacteria.

The studies compared ultrasound and carbon fiber curettes; curettage with or without antibiotics; conventional scaling and the Er:YAG laser.

##### Mechanical treatments

Karring et al. (2005) compared the treatment results obtained with the Vector^®^ ultrasound system and with carbon fiber curettes. After 6 months of follow-up, no significant differences were found between the two techniques, and neither proved sufficient to treat peri-implantitis. Same results were obtained by Persson et al. (2010) with titanium curettes and with ultrasonic device. After 6 month of follow up, no differences were found to reduce microbiota neither proved sufficient to treat peri-implantitis.

The study conducted by Sham et al. (2011) compared mechanical debridement using carbon curettes and antiseptic therapy (MDA) with amino acid glycine powder (AAD). After 6 months of follow up treatment both study group resulted in limited clinical attachment level and the bleeding was reduced in AAD group as compared to MDA group.

 Schwarz et al. (2006b), Renvert et al. (2011) and Persson et al. (2011) compared the Er:YAG laser with air abrasive device. The author recorded limited improvement in clinical parameters in both the group but the bacterial count was not reduced after 6 month of follow up.

##### Mechanical treatments associated to antibiotics

The two studies of Renvert et al. (2006, 2008) published in the year 2006 and 2008 evaluated the treatment in 32 patients, comparing local minocycline microspheres and chlorhexidine gel debridement. After 1 year of treatment both study group showed improvement in plaque index, pocket depth and bleeding without improvement in terms of microbiota. In relation to bacterial load, there were no differences in the change in bacterial composition in the two groups after treatment and further studies were needed to establish how often such treatment must be repeated. Similarly Schar et al. (2013) examined the benefit of photodynamic therapy (PDT) over minocycline microspheres. In both group significant reductions in mucosal inflammation was observed up to 6 month.

The studies published by Hallstrom et al. (2012) in 2012 had used systemic antibiotic azithromycin for 4 days. After 6 months of follow up, there was improvement only in oral hygiene but this study could not provide evidence.

Machtei et al. (2012) evaluated and compared the matrix chips (MatrixC) with that of chlorhexidine chips (PerioC) in 60 patients with probing depth 6–10 mm and bone loss >2 mm. The results yields after 6 month of repeated treatment shows probing depth reduction was greater in the PerioC (2.19 ± 0.24 mm) compared with MatrixC (1.59 ± 0.23 mm). Half in both groups reduced bleeding on probing. Clinical attachment level gains for both groups were significant. However, to fully appreciate mechanism of this treatment, a further study is needed.

#### Surgical techniques

Surgical treatment of peri-implantitis lesions may be performed in cases with considerable pocket formation (larger than 5 mm) and bone loss. Surgical techniques can be divided into resective and regenerative surgery. These techniques is used depending upon the type of bony defects whereas Schwarz et al. (2014) have demonstrated that combined surgical procedure is effective in controlling advanced peri-implantitis lesion.

Aghazadeh et al. (2012) concluded that resective surgical procedures coupled with bovine derived xenograft and placement of collagen membrane have more radiographic evidence of bony defect filled as compared to autogenous bone graft.

The 2-years result by Schwarz et al. (2008) demonstrated that both nanocrystalline hydroxyapatite and application of the combination of natural bone mineral and collagen membrane were efficacious in providing clinical significant reduction of the pocket probing depth and gain in clinical attachment level but in the 4 year study of Schwarz et al. (2009) application of the combination of natural bone mineral and collagen membrane were more efficacious in clinical improvement as compared to nanocrystalline hydroxyapatite. But the 6 months of Schwarz et al. (2006a) study concluded the application of nanocrystalline hydroxyapatite and guided tissue regeneration showed significant improvement in clinical parameters.

Wohlfahrt et al. (2012) evaluated the 12 months outcome by adding porous titanium granules (PTG) together with an open flap procedure and in conjunction with mechanical debridement of the implant surface for decontamination with 24 % ethylenediaminetetracetic acid gel followed by antibiotics (amoxicillin and metronidazole) 3 days prior to surgery and for 7 days after surgery. Both the treatment demonstrated significant improvements in probing pocket depth but the reconstruction with PTG resulted in better radiographic peri-implant defect fill.

Romeo et al. (2007) have compared the efficacy of resective surgery with that of implantoplasty. The results obtained after 3 years of therapy demonstrated that the marginal bone loss was significantly lower after implantoplasty.

Schwarz et al. (2011, 2012) in two studies (2011 and 2012) of advanced peri-implantitis evaluated and compared the efficacy of Er:YAG laser (ERL) surface debridement/decontamination (DD) with that of plastic curettes and cotton pellets (CPS) soaked in sterile saline and both procedure were followed by an implantoplasty at the exposed implant surface and were augmented with a natural bone mineral and covered with a collagen membrane. After 24 months of treatment, CPS group yield significant reduction in bleeding on probing and the radiographic bone fill at the intra-bony defect were same in both groups but the clinical attachment values were not significantly different in both groups.

The study by de Waal et al. (2013) demonstrated that the adjunctive benefits derived from the addition of resective surgical treatment consisting of apically re-positioned flap, bone re-contouring and surface debridement and with 0.12 % CHX + 0.05 % CPC to a placebo-solution (without CHX/CPC) tend to be greater immediate suppression of anaerobic bacteria on the implant surface than a placebo-solution, but does not lead to superior clinical results.

## Discussion

The treatment protocol differs depending upon whether it is peri-implant mucositis or peri-implantitis. Until now, no particular treatment protocol has been shown effective. There are number of treatment protocol for the resolution of diseases. But this study highlighted that diseases resolution is satisfactory by surgical treatment. Peri-implant mucositis can be treated by non-surgical treatment (Schar et al. 2013). If the peri-implantitis is diagnosed then the treatment protocol depends on the intraosseous defect. If the bony defect is minimum then implantoplasty can improve the bony defect (Romeo et al. 2007).

Non-surgical treatment could improve significant clinical parameters but bacterial pathogens are not reduced. Treatment standard of peri-implantits can be improved by decreasing the bacterial pathogen hence it is effective if resective surgery is followed in the incipient case of peri-implantitis as well.

In the advanced peri-implantitis combined treatment of resective and regenerative surgical procedure followed by surface decontamination yields good osseo-integration (Schwarz et al. 2012). de Waal et al. (2013) study concluded that surface decontamination/debridement reduce bacterial count but there was no superior improvement in clinical parameters hence guided bone regeneration (Aghazadeh et al. 2012) or the application of bone substitute (Schwarz et al. 2009) (nanocrystalline hydroxyapetite) can be efficacious for the treatment of peri-implantitis. The majority of surgical protocols include pre-operative or post-operative systemic antibiotics followed by post-operative chlorhexidine rinse. Maintenance phase after surgery is also important which include oral hygiene instructions and surface biofilm removal.

Although we performed a comprehensive analysis of the effects of surgical and non-surgical treatment, there were some limitations to this systematic review. First, our systematic review could not provide the implant survival rate because of insufficient eligible information. Second, high quality study with survival rate was not there which may compromise our conclusion. There could be potential language bias in this systematic review as we only considered literature written in English.

## Conclusions

Complete osseointegration is difficult to achieve. Even though the different treatment modalities cannot be comparable, however the outcome of surgical treatment of peri-implantitis is good. Surgical procedures for peri-implantitis in human have shown positive results but long-term study is needed to achieve the reliability of the treatment.
